# Editorial: Peptide-based immunotherapy against emerging viral infections

**DOI:** 10.3389/fimmu.2023.1169034

**Published:** 2023-03-01

**Authors:** Shisong Jiang

**Affiliations:** Department of Oncology, University of Oxford, Oxford, United Kingdom

**Keywords:** emerging viral infections, immunotherapies, epitope mapping, T cell immunity, apoptosis, viral entry inhibition, peptide-based vaccines

Emerging viral infections like COVID-19, Ebola, Zika, SARS, and MERS are of great global health concern as they can cause pandemics, severe illness, and death. The development of vaccines and treatments are crucial in combating epidemics and minimizing their impact (1).

Peptide-based immune therapies are a promising approach for the prevention and treatment of emerging viral infections. These therapies utilize short peptides, either to stimulate the body’s immune system to eradicate the pathogens; alternatively, directly kill or inhibit the virus. The rapid design and production of peptides, as well as their ability to target specific viral antigens and enhance the immune response, make them a valuable tool for treating newly emerging viruses.

In this Research Topic, we have collected several articles that highlight the potential of peptide-based immune therapies for treating emerging viral infections. These articles mainly focus on epitope screening and epitope-based vaccine design, as well as peptide-based inhibition of viral entry and transmission.

T-cell epitope -based vaccines aim to activate CD4+ and CD8+ T-cells presented by antigen-presenting cells (APCs). CD4+ T cells are activated by epitopes presented by MHC class II molecules on the surface of APCs, helping with antibody production and CD8+ T cell activation. In contrast, epitopes presented by MHC class I molecules on APCs activate CD8+ T cells, which function as killer cells to destroy infected cells that express the same viral peptides on the surface of target cells. To design epitope-based vaccines, mapping T-cell epitopes for MHC binding is crucial. A traditional approach involves using synthetic overlapping peptides (SOPs) that cover the entire length of a target viral protein. SOPs serve as a peptide library for screening epitopes that bind to MHC molecules. However, identifying epitopes this way can be a time-consuming and labour-intensive process due to the significant polymorphism of MHC molecules (2–4).

An alternative approach to mapping T-cell epitopes is to use computer algorithms to predict them. However, the accuracy of predicted epitopes is often suboptimal, and experimental validation is necessary to confirm the binding affinity of predicted epitopes to MHC molecules. In their review article, Sun et al. focus on improving the accuracy of algorithm methods for in silico prediction of T cell epitopes. Typically, the accuracy of a single prediction analysis using conventional in silico prediction is only 50%-70%. To improve accuracy, the concept of “integration” is proposed, which involves using multiple analysis algorithms for an identical antigen. The dominant epitopes predicted by each tool can be selected, and the intersection of multiple tools is used to define the dominant epitopes. Additionally, the results of multiple analyses can be compared to validate the bioinformatics-level results. While the “integration” method is an interesting new idea, its practicality and reliability needs to be tested over time. One caveat is that if different algorithms adopt the same or similar principles, such as those based on MHC binding affinity, the power of cross-validation by two or more principle-same algorithms may be reduced. Regardless of the methods used, the final validation should be performed through wet lab experiments.

As an example of an epitope-based vaccine estimated in silico, Suleman et al. aimed to develop a multiepitope subunit vaccine against the monkeypox virus. This zoonotic virus can cause human infections and produces symptoms similar to smallpox. To identify potential epitopes, the authors used a combination of computational and molecular modelling techniques on various monkeypox virus proteins. Using immunoinformatic tools, the authors designed a multiepitope subunit vaccine consisting of several potential epitopes from different monkeypox virus proteins. They then conducted molecular dynamics and immune simulations to assess the vaccine’s stability and immune response. While the vaccine design and testing can be performed in silico, it is purely hypothetical and far from even preclinical validation. Nevertheless, it represents a small step in the long journey to develop an effective vaccine.

In addition to stimulating the immune system, peptides can also directly kill or inhibit viruses. This can be achieved through several mechanisms, including direct inhibition of receptor binding, viral replication, induction of viral lysis, and induction of apoptosis in infected cells. The combination of these mechanisms makes peptide-based therapies a powerful tool for fighting viral infections.

The study by Ohradanova-Repic et al. focuses on the use of lactoferrin-derived synthetic peptides and lactoferricin, a naturally occurring antimicrobial peptide, as a potential treatment for COVID-19. The authors investigated the ability of peptides to block TMPRSS2-mediated priming of the SARS-CoV-2 virus, a key step in the process of virus entry into host cells.

The results of the study suggest that lactoferricin and synthetic peptides derived from lactoferrin may have potential as a therapeutic agent for COVID-19, as it can inhibit a key step in the virus entry process. Further research is needed to investigate the efficacy and safety of these peptides as a treatment for COVID-19. This study provides evidence that peptides has the potential to block a key step in the SARS-CoV-2 virus entry process, and may be promising candidates for potential treatment for COVID-19.

Chen et al. investigated the effects of two peptides derived from tumor necrosis factor (TNF) on HIV-infected cells. They had previously identified these peptides, known as P13 and P16, as being responsible for inducing apoptosis and necrosis, respectively. The researchers found that treating HIV-infected MT2 cells with the P13 peptide, which induces apoptosis, reduced the viral load (measured by the percentage of cells positive for the HIV p24 antigen) in a dose-dependent manner. Specifically, the researchers found that at a concentration of 50 µM, P13 reduced the percentage of p24-positive cells by approximately 40% compared to untreated cells. In contrast, treatment with the P16 peptide, which induces necrosis, did not significantly affect the viral load. These findings suggest that inducing apoptosis in HIV-infected cells using the P13 peptide could be a potential strategy to reduce viral load in HIV-infected individuals.

In conclusion, peptide-based immune therapies are a valuable tool for the treatment of emerging viral infections. Their ability to stimulate the immune system and directly kill or inhibit viruses, as well as their rapid design and production, make them a promising approach for the treatment of newly emerging viruses. While more research is needed to fully understand their potential, the use of peptide-based immune therapies holds great promise for improving global health outcomes.

## Author contributions

The author confirms being the sole contributor of this work and has approved it for publication.
